# Factors Limiting Radial Growth of Conifers on Their Semiarid Borders across Kazakhstan

**DOI:** 10.3390/biology12040604

**Published:** 2023-04-16

**Authors:** Nariman B. Mapitov, Liliana V. Belokopytova, Dina F. Zhirnova, Sholpan B. Abilova, Rimma M. Ualiyeva, Aliya A. Bitkeyeva, Elena A. Babushkina, Eugene A. Vaganov

**Affiliations:** 1 Department of Biology and Ecology, Toraighyrov University, Pavlodar 140008, Kazakhstan; ualiyeva.r@gmail.com (R.M.U.); aliya_bit@mail.ru (A.A.B.); 2 Khakass Technical Institute, Siberian Federal University, 655017 Abakan, Russia; dina-zhirnova@mail.ru (D.F.Z.); babushkina70@mail.ru (E.A.B.); 3 Department of Microbiology and Biotechnology, S. Seifullin Kazakh Agrotechnical University, Astana 010011, Kazakhstan; sholpana_jan@mail.ru; 4 Institute of Ecology and Geography, Siberian Federal University, 660036 Krasnoyarsk, Russia; eavaganov@hotmail.com; 5 Department of Dendroecology, V.N. Sukachev Institute of Forest, Siberian Branch of the Russian Academy of Science, 660036 Krasnoyarsk, Russia

**Keywords:** conifers, tree-ring width, Kazakhstan, lower forest boundary, climate–growth relationship

## Abstract

**Simple Summary:**

In the dry and hot climates of Central Asia, forested areas are small and vulnerable to climate change. Therefore, it is important to understand the reactions of tree growth to climatic factors there. We studied several habitats of Scots pine, Schrenk spruce, and Zeravschan juniper near the semiarid limits of the respective forest types across Kazakhstan, the largest country in Central Asia. Standardized chronologies of tree-ring width were obtained for each site from wood samples, and then compared among themselves with a series for temperature and precipitation. Large distances and differences between species and habitats limited similarity in the dynamics of conifer growth. However, a common pattern was found in their reactions to climate. Maximal temperatures of the current and previous growing seasons were found to be crucial factors limiting the growth of all considered forest stands. Reactions to the precipitation and drought index are positive, but their strength depends on the species and aridity of a particular habitat. Temporal intervals of climatic impact on tree growth also vary across the country.

**Abstract:**

The forests of Central Asia are biodiversity hotspots at risk from rapid climate change, but they are understudied in terms of the climate–growth relationships of trees. This classical dendroclimatic case study was performed for six conifer forest stands near their semiarid boundaries across Kazakhstan: (1–3) *Pinus sylvestris* L., temperate forest steppes; (4–5) *Picea schrenkiana* Fisch. & C.A. Mey, foothills, the Western Tien Shan, southeast; (6) *Juniperus seravschanica* Kom., montane zone, the Western Tien Shan, southern subtropics. Due to large distances, correlations between local tree-ring width (TRW) chronologies are significant only within species (pine, 0.19–0.50; spruce, 0.55). The most stable climatic response is negative correlations of TRW with maximum temperatures of the previous (from −0.37 to −0.50) and current (from −0.17 to −0.44) growing season. The strength of the positive response to annual precipitation (0.10–0.48) and Standardized Precipitation Evapotranspiration Index (0.15–0.49) depends on local aridity. The timeframe of climatic responses shifts to earlier months north-to-south. For years with maximum and minimum TRW, differences in seasonal maximal temperatures (by ~1–3 °C) and precipitation (by ~12–83%) were also found. Heat stress being the primary factor limiting conifer growth across Kazakhstan, we suggest experiments there on heat protection measures in plantations and for urban trees, alongside broadening the coverage of the dendroclimatic net with accents on the impact of habitat conditions and climate-induced long-term growth dynamics.

## 1. Introduction

Forests are vital for the existence of humankind and the entire biosphere of our planet. In addition to timber and other resources, they provide habitats for a multitude of plant and animal species, play an important role in the protection and regulation of water resources and prevention of soil erosion, and are a crucial part of the carbon cycle [1,2,3,4]. The consequences of current climatic trends for forest vegetation vary greatly depending on a number of factors, such as local conditions (moisture availability, orography, etc.), ecophysiological characteristics of species, individual genotypes, and even tree age [5,6,7,8,9,10,11,12]. Even the direction of impact is ambiguous: in some regions, there is a strong negative or even catastrophic effect; in others, stable forest growth or even positive changes can be observed [13,14,15,16,17,18]. Recent warming, often accompanied by a drop in precipitation, and the increased severity and frequency of climatic extremes have been leading to shifts in vegetation distribution ranges toward higher latitudes and longitudes [19,20,21].

From this perspective, such regions as Central Asia are of particular importance, where the spatially synchronous long-term trend of temperature increase has been noted over the past century, significantly exceeding the global warming rate, especially during the winter [22,23,24,25,26]. Simultaneously, a sharp spatial heterogeneity was observed in the dynamics of precipitation and various drought indicators [22,25,26,27,28,29,30]. Uncertainty also exists in future climate scenarios for water availability in this region [22,24,25,26]. We can assume that in Central Asia, the impact of human activity on the local and regional climate may prevail over global trends. One of the vivid examples of anthropogenic interference is the Aral Sea crisis due to widespread irrigation, which led to an increase in climate continentality (the seasonal magnitude of temperature) and shifts in the seasonal distribution and amount of precipitation in the surrounding area [22,31]. The consequences of long-term climate trends for the vegetation of this large region are also ambiguous [32,33,34]. In particular, climate change may affect the growth of forests confined mainly to mountainous areas, which is especially important for biodiversity conservation due to the small and fragmented forested areas and the large number of endemic and endangered species in Central Asia [35,36].

Kazakhstan is one of the important territories of Central Asia, with its vast area and a wide range of natural and climatic zones due to orographic diversity and geographical location. This makes it a convenient testing range for studying the climatic reactions of vegetation. However, forest ecosystems in Kazakhstan occupy relatively small areas. According to state statistics data (https://ecogosfond.kz/ltty-bajandama/, accessed on 10 February 2023), the total area covered by shrubs and forests was 15.7 million ha at the end of 2021 (5.8% of the country’s territory); however, semi-desert saxaul forests account for almost half of this. The remaining forest stands are located in several areas: Altai and Tien Shan mountains, the northern forest steppe, tugai forests along large rivers, and agricultural forest belts [37,38,39]. Small fragmented areas and rigid confinement to landscape make the forest ecosystems of this region vulnerable to climate change and, at the same time, crucially important for biodiversity conservation. Therefore, beginning from the middle of the last century, Kazakhstan began pursuing a policy of conservation, restoration, and sustainable use of forests [37,40,41,42,43,44]. As a result, significant positive trends were recorded. For example, from 2010 to 2021, the forested area increased by 11%, while timber stock increased by 20% (https://stat.gov.kz/official/industry/157/statistic/7, accessed on 10 February 2023). The territory of protected natural areas, including forest ones, has also increased in Kazakhstan recently (https://ecogosfond.kz/ltty-bajandama/, accessed on 10 February 2023).

Within the framework of sustainable forestry management and conservation of biodiversity in Kazakhstan under climate change, understanding the climate responses of forests becomes especially important. One of the promising areas of research in this regard, which helps understand and predict the impact of climate fluctuations and trends on the productivity of trees and dynamics of forest ecosystems, is the analysis of climate–growth relationships for key species based on their tree rings [45,46,47,48,49]. Unfortunately, dendroclimatic studies in the country are irregular, the number of published works is small, and many of them are not easily accessible (see review by Zubairov et al. [38] and reference list there). Therefore, filling the “blind spots” in the dendroclimatology of the region is a priority. In this study, on the example of several conifer species, the climatic reactions on the semiarid border of forest ecosystems of Kazakhstan are analyzed along a large-scale geographic transect from the northern forest steppes to mountain subtropical forests in the south of the country. We hypothesized that there are two main components in the growth response of these conifers to the conditions of the growing season: (1) variable response to the moisture regime (precipitation) depending on local conditions, and (2) stable limitation by high temperatures throughout the transect.

## 2. Materials and Methods

### 2.1. Study Region and Sampling Sites

The Republic of Kazakhstan has a complex and diverse landscape. The southwest, north, and central parts are characterized by flat terrain at 200–300 m a.s.l. In the east and southeast, there are mountain ranges reaching 5 to 6 thousand m a.s.l. Natural zones range from deserts and steppes on plains, to mountain broad-leaved, mixed, and coniferous forests, and to tundra and bare rocks. The climate of Kazakhstan is sharply continental, with hot summer, cold winter, and large daily temperature fluctuations [50]. In the latitudinal direction from north to south, there is a gradual transition from the temperate climatic zone, with a maximum of precipitation in July and a minimum in the winter months, to the subtropical zone, with a maximum of precipitation in spring and an extremely dry second half of the summer.

**Figure 1:**
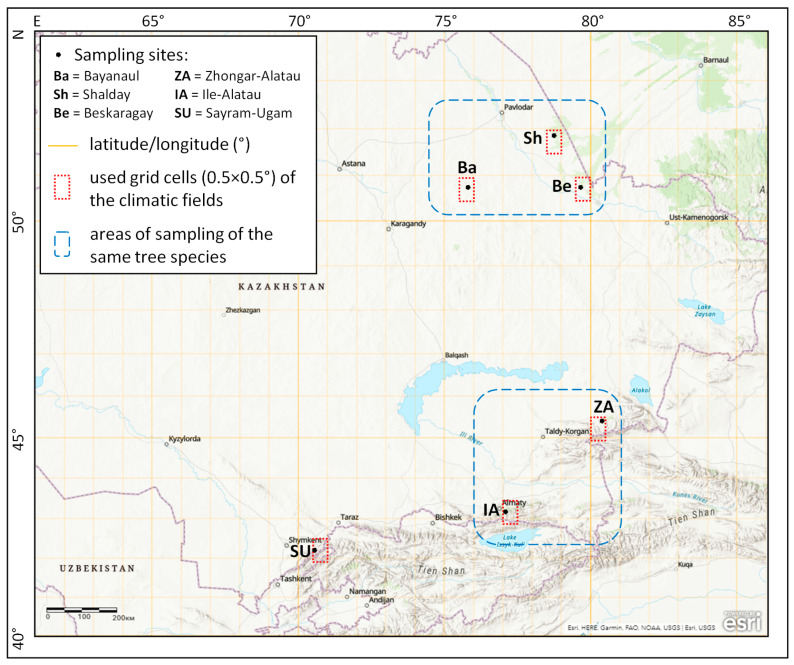
The study area. Markers represent sampling sites (Ba—Bayanaul, Sh—Shalday. Be—Beskaragay, ZA—Zhongar-Alatau, IA—Ile-Alatau, SU—Sairam-Ugam); small dotted rectangles represent geographic grid cells 0.5 × 0.5° for which climatic series from spatially distributed fields were used in correlation analysis; dashed rectangles mark sites where the same tree species were sampled. The map was created in the ArcGIS tool (https://www.arcgis.com/home/webmap/viewer.html; accessed on 10 April 2023) © Esri.

**Figure 2:**
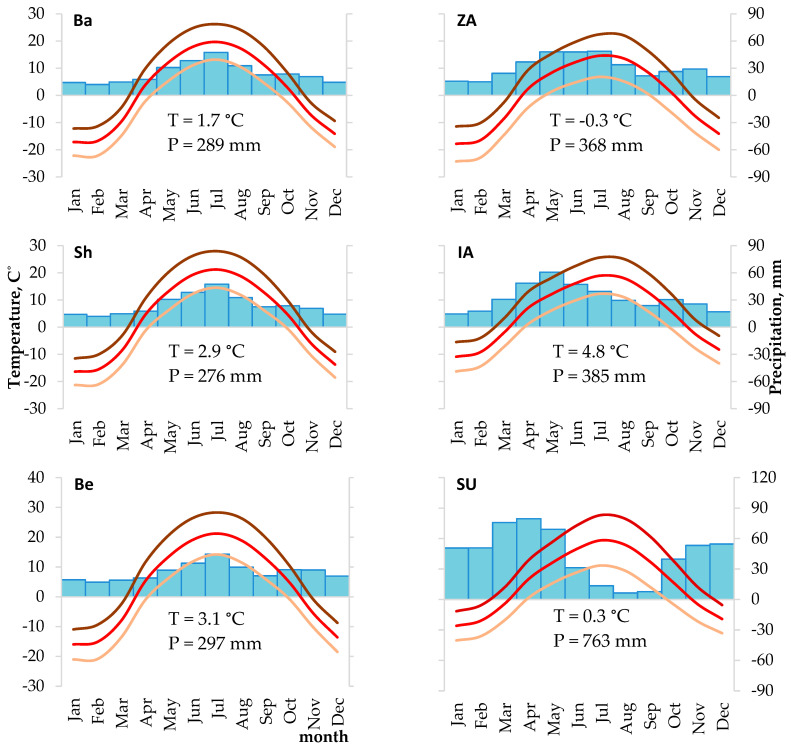
Average monthly climatic characteristics (Climatic Research Unit Time Series, CRU TS 1901–2020) for geographic grid cells where sampling sites are located: Ba—Bayanaul, Sh—Shalday, Be—Beskaragay, ZA—Zhongar-Alatau, IA—Ile-Alatau, and SU—Sairam-Ugam. Lines represent maximum (dark), mean (medium), and minimum (light) temperatures; bars represent precipitation. Numbers represent the average annual mean temperature (T) and the average annual sum of precipitation (P). Note that at sampling sites located in mountain foothills (ZA, IA, and SU), temperatures are probably higher than the values for mountainous areas that are presented here (cf. Figure A2).

**Figure A1:**
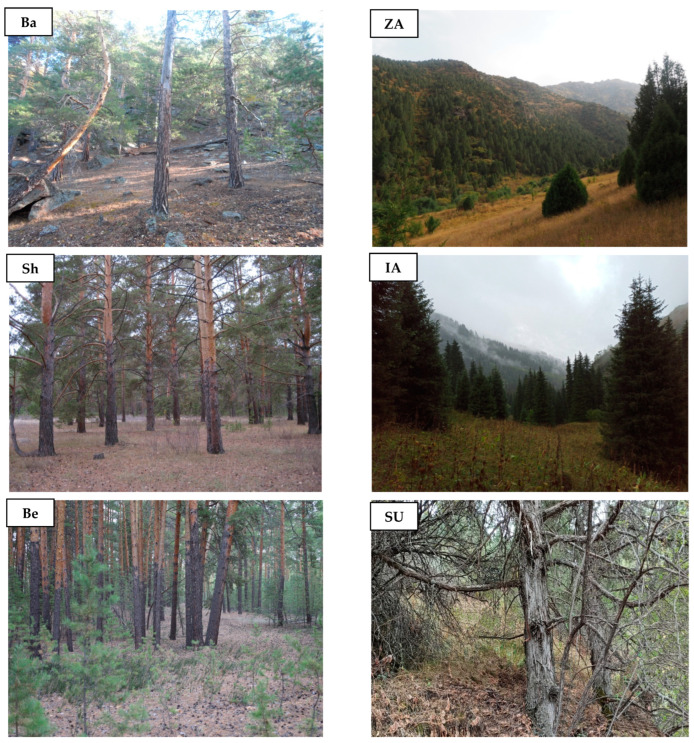
The sampling sites (Ba—Bayanaul, Sh—Shalday, Be—Beskaragay, ZA—Zhongar-Alatau, IA—Ile-Alatau, and SU—Sairam-Ugam).

In 2017–2019 in the territory of Kazakhstan, six dendrochronological sampling sites were established in conifer forest stands within protected areas along a vast geographic transect (Figure 1, Table 1). Spatially, these sites can be divided into three groups in accordance with natural zones, climatic conditions (Figure 2), and forest-forming coniferous species. Three sites (Bayanaul, Shalday, and Beskaragay) are located in isolated pine forests (*Pinus sylvestris* L.) surrounded by steppes in a temperate climate. The Bayanaul site in the Bayanaul State National Natural Park (SNNP), part of the Kazakh Highlands, is located on a gentle (up to 10°) southeastern slope, in the pure pine stand on stony and underdeveloped chestnut soils without grass cover (Figure A1). Pine forests in this area begin at 450–550 m a.s.l., depending on the slope aspect, and reach mountain tops at 1026 m a.s.l. The Shalday (Ertis-Ormany State Forest Nature Reserve, Shaldaysky Branch) and Beskaragay (Semey-Ormany State Forest Nature Reserve, Kanonersky Branch) sites are located on plains in the isolated pine forests surrounded by dry steppes. There, the spatial distribution of forests is regulated by soil type and the underground water table rather than by elevation; thus, these areas can be considered the southern fringe of the pine distribution range. The soils within these forests are sandy; the grass cover is sparse. At the Beskaragay site, higher moisture is observed because groundwater is closer to the surface.

The southeastern group of sampling sites (Zhongar-Alatau and Ile-Alatau) is represented by spruce forests (*Picea schrenkiana* Fisch. & C.A. Mey) in the low montane zone of the eponymous ranges within the Western Tien Shan Mountains. They are located in the transition zone between temperate and subtropical climates. Spruce in these mountains is distributed on average from 1100–1200 to 3600 m a.s.l. In Zhongar-Alatau SNNP, the sampling site was selected in a pure spruce forest with a sparse grass-forb cover on forest chernozem soil at the foot of the northern slope. The sampling site in the Ile-Alatau SNNP is similar to Zhongar-Alatau but more humid, located in the river valley at a distance of about 4 km from the lower forest boundary horizontally and ~250 m above. The herbaceous vegetation of this area is represented by more moisture-sensitive species, including ferns.

The southernmost sampling site in the Sairam-Ugam SNNP is located in the juniper forest belt (*Juniperus seravschanica* Kom., sometimes considered as ssp. or var. of *Juniperus polycarpos* K.Koch or *Juniperus excelsa* M.Bieb.), which is bordered with broad-leaved trees down the slope (for example, walnut *Juglans regia* L.), and is situated on the Talas Alatau Ridge of the Western Tien Shan Mountains, where the climate is subtropical. The site was selected on the northeastern slope (10–15°) of a river valley, on stony mountain forest soils, sparse herbaceous vegetation is represented by steppe forb species. The total vertical distribution area of the species is 800 to 3200 m a.s.l., but its lower boundary varies from 800 to 2000 m a.s.l. depending on the area and slope.

All sampling sites were established close to the lower boundary of the distribution for respective conifer species, but their elevation increased from 170 to 2000 m a.s.l. from north to south. In the northern group, the distance between the Shalday and Beskaragay sites is about 80 km, and Bayanaul is located 240–260 km from them. The sites of the southeastern group are located more than 600 km from the northern group and at a distance of 340 km from each other. The southernmost site is located at a distance of more than 1000 km from the northern group and more than 500 km from the southeastern group.

### 2.2. Data and Their Analysis

Wood samples (cores) were taken from dominant/subdominant mature healthy trees without close neighbors with an incremental borer at the height of ~1.3 m from the eastern or western side of the tree trunk, on plain ground, and perpendicular to the slope aspect of the mountains. Samples for further research were prepared according to the generally accepted method [51]. The tree-ring width (TRW) was measured on a LINTAB tool using the TSAPWin software [52], and the resulting series were cross-dated with verification in the COFECHA software [53]. The standardization of individual tree-ring chronologies was carried out using the ARSTAN program [54], where age trends were described by exponential or linear functions and removed. Then, the radial growth indices of individual trees were converted by the bi-weighted average into generalized local standard chronologies characterizing the common growth variability in each investigated site. Chronologies were evaluated according to the following statistical characteristics: standard deviation *SD*, mean sensitivity coefficient *sens* (the ratio of the modulus of the successive values’ difference to their mean, averaged over the entire length of the chronology), first-order autocorrelation *ar*_−1_, mean inter-series correlation coefficient *r-bar*, and expressed population signal *EPS* [51,55]. The development of averaged chronologies and the calculation of their statistical characteristics were also performed in ARSTAN. Before dendroclimatic analysis, the beginning of chronologies was cut off based on the criterion of being poorly supplied with samples and therefore having 40-year *EPS* < 0.85 [55].

The climate response of TRW was estimated by Pearson correlation coefficients of local chronologies with monthly series of mean, maximum, and minimum temperatures, and the sum of precipitation interpolated for the corresponding geographical coordinates of the spatially distributed field Climatic Research Unit Time Series (CRU TS, 1901–2020), which is publicly available in the Climate Explorer database (https://climexp.knmi.nl/start.cgi, accessed on 10 February 2023) of the Royal Netherlands Meteorological Institute (KNMI). Correlations were calculated for each month from June of the previous year to September of the current year. By an exhaustive search of the longer time intervals for generalizing the climatic variable (taking into account intervals with the same sign of TRW correlations with monthly series), seasonal climatic factors were obtained that have the most pronounced effect on the radial growth of conifers (maximum correlations). Additionally, the Standardized Precipitation Evapotranspiration Index (SPEI) over 1901–2018, available in the same database (https://climexp.knmi.nl/start.cgi, accessed on 11 April 2023), was selected as drought indicators, and correlations with TRW chronologies were calculated for its 12-month series in the respective geographic grid cells.

As pointer years, five years of the highest growth indices (positive) and five years of the lowest growth indices (negative) were considered at each site for the common period between chronology and the climatic series. Climatic deviations of pointer years were estimated for seasonal climatic factors by comparing their mean values and range of variability (*mean ± SD*) between sets of the positive and negative pointer years.

The significance level *p* of the correlation coefficients and of the differences between positive and negative pointer years was assessed by a two-tailed *t*-test. Correlation analysis and comparison of means were performed in Microsoft Excel 2007 (Microsoft Corporation, Redmond, WA, USA).

## 3. Results

### 3.1. Developed Chronologies and Their Statistic Characteristics

**Figure 3:**
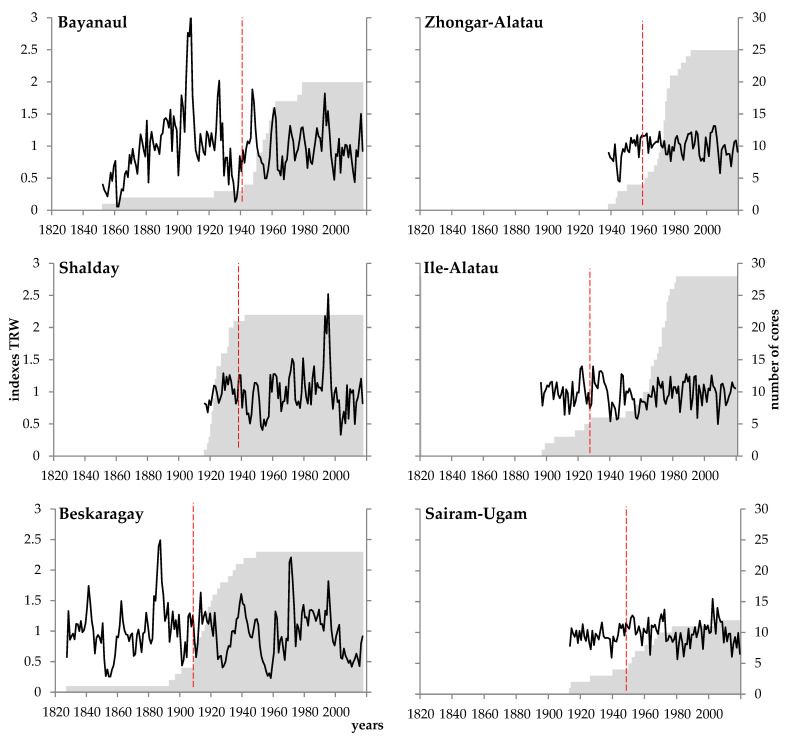
Conifer growth dynamics. Line represents standard local chronology; shaded area represents sample depth (number of cores for each year); and vertical dashed line shows the first year of *EPS* ≥ 0.85, i.e., beginning of period suitable for dendroclimatic analysis.

The statistical characteristics of the developed chronologies are given in Table 2. The total sample size for all sites was 132 cores, from 12 to 29 cores per site. The total lengths of the chronologies vary from 82 to 191 years, and the periods suitable for dendroclimatic analysis (*EPS* ≥ 0.85) vary from 57 to 105 years. The cambial age of the oldest trees (the maximum length of the measured series from bark to pith) is 102–191 years, 124 years, and 107 years for pine, spruce, and juniper, respectively (Figure 3). The average tree-ring width is 1.2–2.5 mm; the slowest growth was recorded for pine at the Beskaragay site. Pine radial growth has the highest variability, while for spruce and juniper, this characteristic is more limited both in general (standard deviation) and its inter-annual component (mean sensitivity coefficient). The common external signal is strong in pine and spruce chronologies, but more moderate in the TRW of juniper. Significant first-order autocorrelation is observed for all sites and species, but it is also maximum for pine and minimum for juniper.

To assess a possible regional signal in the chronologies, a comparative correlation analysis of the chronologies was performed (Table 3). Correlations between chronologies depend on the between-site distance. For the Shalday site, there is a good connection with other northeastern sites, Bayanaul and Beskaragay, but the chronologies of these two (more distant) sites weakly correlate with each other. In the southeast, the TRW chronologies of spruce in the Zhongar-Alatau and Ile-Alatau sites have a significant relationship with each other. Their correlations with pine chronologies in the north are positive, but insignificant (up to 0.2). In the southernmost Sairam-Ugam site, the radial growth of juniper does not correlate with the growth of conifers in other areas.

### 3.2. Climatic Response of Conifer Radial Growth

**Figure 4:**
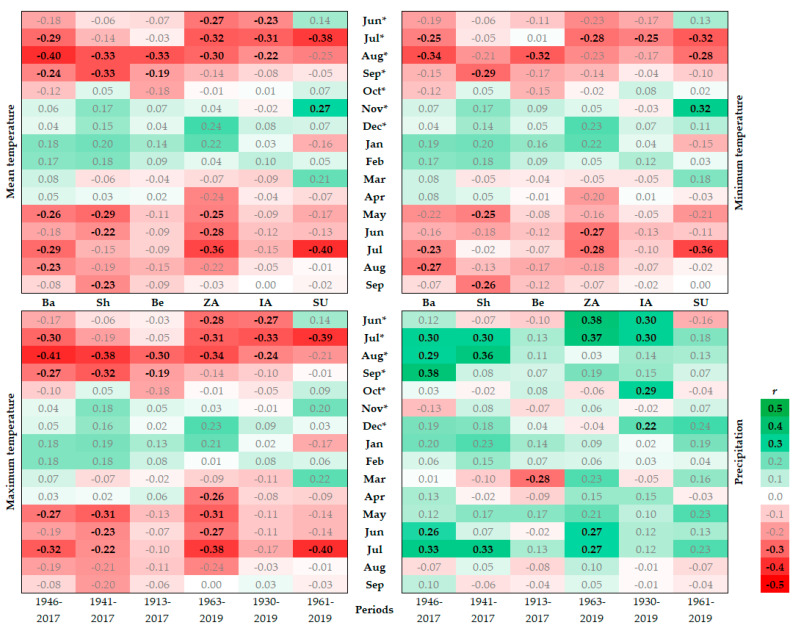
Correlation coefficients of local standard tree-ring chronologies (B—Bayanaul, C—Shalday. Be—Beskaragay, ZA—Zhongar-Alatau, IA—Ile-Alatau, and SU—Sairam-Ugam) with monthly series of precipitation, maximal, mean, and minimal temperatures from previous June to current September. Color gradient marks correlation values (green, positive; red, negative; see legend). Correlations marked with bold black font are significant at *p* < 0.05. Asterisks (*) mark months of the previous year.

Figure 4 presents the correlation coefficients of the TRW indices for all sampling sites with monthly climatic series. For the entire study region and the range of habitat conditions, there is a general pattern of a negative relationship between tree radial growth and temperature (stronger with the maximum one) and a positive relationship between TRW and precipitation. Both reactions were observed approximately within the summer and early autumn of the previous year and spring-summer of the current year, i.e., previous and current growing season. However, the intensity and seasonality of this response vary greatly depending on the species and geographic location.

At the Bayanaul site, a significant negative relationship between pine growth and temperatures during the previous year is recorded in July–September for mean and maximum temperature, and in July and August for minimum temperature. The temperature during the current year significantly affects pine growth in May, July, and August (mean), May and July (maximum), and July–August (minimum). A positive relationship between precipitation and pine growth indices was recorded from July to September of the previous year and from June to July of the current year. At the Shalday site, the negative relationship of the TRW with the temperatures during the previous year is significant in August–September (mean and maximum) and September (minimum). During the current year, temperature negatively significantly affects the growth of pine in May–July (mean), April–July (maximum), and May and September (minimum). A significant positive relationship between precipitation and pine growth is observed in July–August of the previous year, but only in July of the current year. At the Beskaragay site, a significant negative relationship between pine growth and temperatures was not observed during the current year, but in the previous year, it was recorded in August–September (maximum, mean) or August (minimum). A negative relationship between pine growth and precipitation was registered in March.

For spruce growth in the Zhongar-Alatau and Ile-Alatau sites, a significant negative relationship with the temperatures during the previous year is observed in June–August (maximum, mean) or July (minimum). The response of growth to temperature in the current year in Ile-Alatau is not significant, and in Zhongar-Alatau, it is observed in April–July (maximum), May–July (mean), and June–July (minimum). Precipitation had a positive effect on the growth of spruce in June–July of the previous year at both sites, in the previous December at the Ile-Alatau site, and in the current June–July at the Zhongar-Alatau site.

At the Sairam-Ugam site, temperature negatively affects the growth of juniper in July (maximum, mean) and July–August (minimum), but positively affects it in November (mean, minimum) of the previous year. In the current year, all temperature series are negatively correlated with the TRW of juniper in July. A significant relationship between the growth of the juniper and precipitation was not observed.

Generalizing the most pronounced reactions of coniferous growth to maximum temperature and precipitation over several months, we identified seasonal intervals in the previous and current years, for which the climatic impact is unidirectional and strongest (Table 4). The annual amount of precipitation was also investigated as an indicator of the water balance of the territory.

The seasonality of the pine response turned out to be similar in the Bayanaul and Shalday sites, differing only for annual precipitation. For the Beskaragay site, there is a shift to a later date of responses to the climate of the previous season, while it was not possible to achieve significant correlations by aggregating series over several months for the total precipitation of any season and the maximum temperature of the current growing season.

Spruce growth in the Zhongar-Alatau site has a more pronounced reaction for all considered seasons, but with a tendency to shift seasonality to earlier dates for the previous and beginning of the current year and to later ones at the end of the current growing season (in comparison to pine). For the Ile-Alatau site, however, the responses to the climate of the current season are not significant, and to the climate of the previous season, they coincide in calendar terms with Zhongar-Alatau for temperature and end later for precipitation.

Juniper in the Sairam-Ugam site significantly reacts to the temperature of both growing seasons and precipitation of the current season, unlike the monthly scale. For annual precipitation in the temperate and transitional climates, there is a latitudinal shift in the seasonality of the response to earlier dates from north to south, but in the subtropical continental climate of the Sairam-Ugam site, seasonality is tied to the severe drought of July–August.

At all sampling sites, conifer radial growth is positively correlated with the annual average SPEI. Maximum correlations were observed during the same annual periods and had similar or higher values as annual precipitation (Table 4).

**Figure 5:**
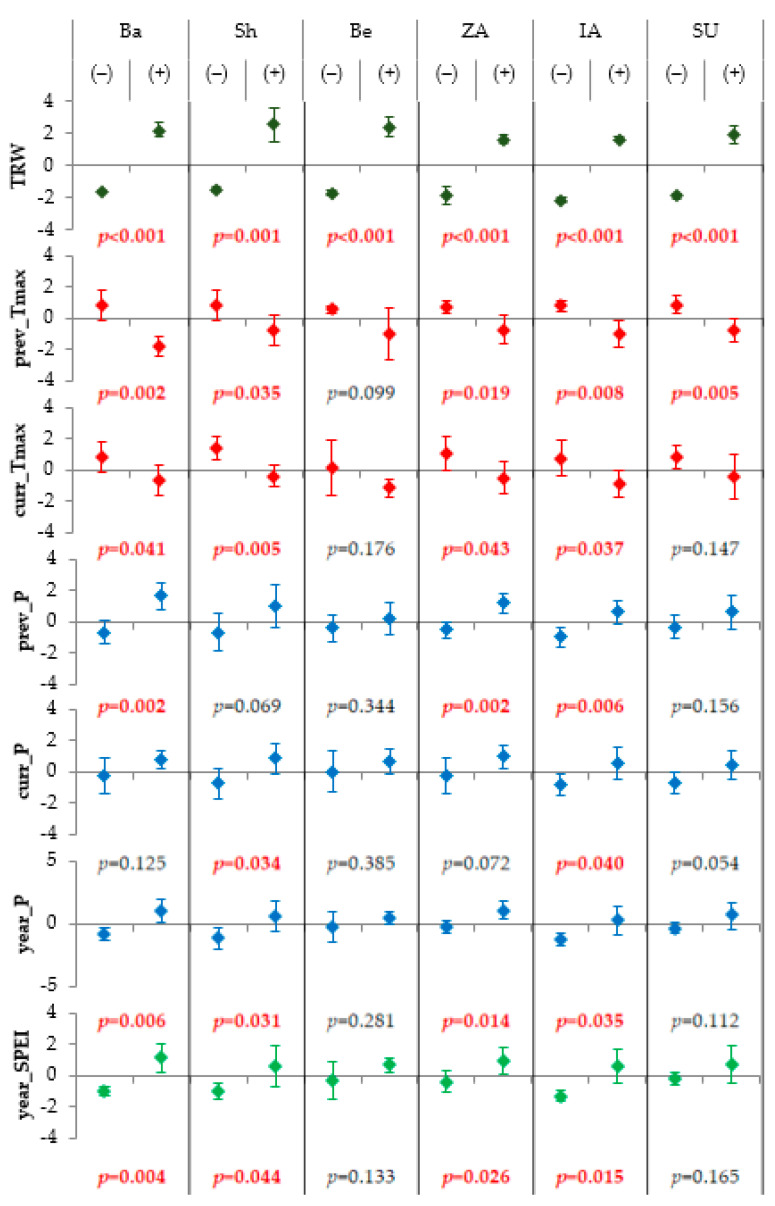
Characteristics of the pointer years, i.e., years with the highest/lowest tree-ring width (TRW) indices at each sampling site (Ba—Bayanaul, Sh—Shalday, Be—Beskaragay, ZA—Zhongar-Alatau, IA—Ile-Alatau, and SU—Sairam-Ugam): mean values (markers) and standard deviations (whiskers) for TRW, average maximal temperature for previous (prev_Tmax) and current growing season (curr_Tmax), sum of precipitation for previous (prev_P) and current growing season (curr_P), annual sum of precipitation (year_P), and average SPEI (year_SPEI). Site-specific intervals of the seasonal generalizing for climatic variables are as presented in Table 4. Marking (−) and (+) represent sets of five negative and five positive pointer years, respectively (years with minimal/maximal values of TRW indices within cover period of climatic series). Presented values of the significance level *p* were calculated for differences in means between negative and positive pointer years (*p* < 0.05 marked by red font). Data points are color-coded by type of variable: TRW, brown; temperature, red; precipitation, blue; SPEI, green. All variables are linearly transformed into *z*-scores (*mean* = 0; standard deviation *SD* = 1).

The analysis of climatic patterns of individual years was carried out by comparing the climate of positive and negative pointer years, i.e., the most favorable years for the conifer growth (maximum standard TRW) and, vice versa, the most stressful years (minimum standard TRW). The difference in the average values of seasonal climatic variables between favorable and stressful years is strictly unidirectional, although it is not significant in all cases. Years with the fast growth of trees are characterized by 1–3 °C lower maximum temperatures of the previous and current growing seasons and more precipitation by 12–83% (Figure 5). The severity of differences tends to be more significant in xeric sites compared to more humid ones, especially for the northern group of sites, where the annual amount of precipitation is less compared to other areas. A certain degree of spatial commonality of pointer years is shown by the fact that maximum radial growth often occurred in the same years at two or three different sites (in 1947, 1970, 1972, 1993, 1995, and 2005, and the coincidence was inter-specific and at large distances for all years except 1995) (Table 5). The same happened for depressions of growth (in 1955, 1956, 1957, and 2012, of which in 1957, the growth of both pine and spruce was suppressed; in other years, depressions were common only within one area/species). On the other hand, cross-directional extremes, i.e., the simultaneous minimum growth of conifers in one area and maximum one in another, occurred only in 1996 and 2003, both times between distant areas.

## 4. Discussion

### 4.1. Spatial Patterns in the Conifer Radial Growth

It has long been noted that in dry and hot regions, the variability of tree-ring chronologies and the common external signal in them (inter-series correlation) synchronously increase from the inner part of the forest zone to trees at the forest boundary, which are under conditions of stronger climatogenic stress [56,57]. However, in the current study, as well as in the work [56], more pronounced differences in statistical characteristics are observed between species of conifers and not between stands of the same species with different moisture availability (i.e., Ba–Sh–Be and ZA–IA in this study). This is especially true for the growth variability of the Scots pine, which is much higher than other species under consideration. We can assume that this may be due to the high phenological plasticity of this species [58], which manifests both in the dynamics of growth under the influence of climate [59,60] and in an extremely wide distribution range and the spectrum of habitat conditions to which *Pinus sylvestris* can successfully adapt [61,62]. Additionally, the observed statistical characteristics are consistent with the suitability of the considered species for dendrochronological studies according to the assessment of [63] if cross-dating of TRW is possible and on what spatial scale. Nevertheless, for all obtained chronologies, the common external signal is sufficient for dendroclimatic analysis.

Spatial correlations between the chronologies of radial growth turned out to be statistically significant only within the species, but here one should take into account the enormous distances within the transect, especially between sampling sites for different species. There can be several main reasons for low correlations:

1. The spatial heterogeneity of the precipitation and moisture regimes, in general, is one of the factors limiting tree growth in semiarid habitats [64,65,66];
2. Inter-species differences in climate response associated with different physiological and morphological adaptation strategies to hot and dry conditions [67,68,69];
3. Phenological differences in periods of active growth between climates and between species [70,71,72];
4. the diversity of local soil landscape and climatic conditions of the habitat (especially in mountain ecosystems), which modulate the dynamics of tree growth even within the same species and climatic zone [73,74,75].

### 4.2. Conifer Growth Limitation by Climatic Factors in the Semiarid Territories of Kazakhstan

The immediate northern neighbor of the steppes of Kazakhstan is South Siberia, where a combination of coniferous/mixed forests in the mountains and steppes on the plains also predetermines the wide distribution of semiarid forest stands of the forest-steppe ecotone and isolated pine forests within the steppe zone. However, despite the similarity in the spectra of natural areas, there are significant differences in the climatic response of pines at the lower/southern forest boundary in Kazakhstan from more northerly populations throughout South Siberia. In the longitudinal transect of the Southern Urals–Altai isolated pine forests–Khakass-Minusinsk Depression–Selenga Highlands, both for pine stands and other tree species forming the semiarid forest boundary, a positive response to seasonal and/or annual precipitation prevails in TRW chronologies, and a negative response to temperature is much weaker in comparison [8,76,77]. Simultaneously, even for the two sites in Kazakhstan closest geographically, Shalday and Beskaragay, located on the southwestern tips of the same Altai pine forests, the negative impact of the temperature during the growing season already seems more pronounced. Moreover, a stable signal for the temperature in the middle of the growing season, primarily the maximum temperature, is also observed for the considered stands of spruce and juniper, regardless of habitat conditions. Comparable correlations of TRW with precipitation and temperature of the growing season or prevalence of the temperature signal were also recorded in other studies of conifer forests in Kazakhstan [78,79,80] and neighboring regions of Central Asia: Uzbekistan, Kyrgyzstan, southwestern Mongolia, and northwestern China [81,82,83,84,85,86,87].

Note that in this study, the strongest response to monthly climatic variables during the growing season is observed to the maximum temperature in all areas, regardless of the species, habitat conditions, and climatic zone. When comparing favorable and stressful years for the growth of conifers, the most stable difference in the climatic regime throughout all transects is also observed for the maximum temperatures of the growing season. We assume that the shift in the priority of tree response from precipitation to temperature is because, in the arid plains of Kazakhstan and other countries of Central Asia (and, accordingly, in the foothills adjacent to them), the temperatures of the growing season reach much higher values than in the corresponding biomes of Southern Siberia due to their location in lower latitudes at comparable altitudes. For example, the average maximum temperature in July, according to the data of meteorological stations in the steppe zone of Siberia (Chelyabinsk, Barnaul, Minusinsk, and Ulan-Ude), is 25–27 °C. In comparison, at the major stations in the Kazakhstan plains (closest to the study areas), these temperatures range from 28 °C (Pavlodar) in the north to 30 °C (Almaty) in the southeast, up to 35.7 °C (Tashkent) in the south. It turns out that if, in the temperate boreal climate of South Siberia, the main factor suppressing tree growth in semiarid ecosystems and determining the position of the forest boundary is water stress, then when moving south toward a subtropical climate, heat stress also begins playing an important role (cf. similar shift of climatic response in China [87]).

Indeed, data from a meta-analysis [88] showed a decrease in the acclimatization potential of evergreen trees to warming temperatures from boreal to tropical biomes, which is associated with higher background temperatures, among other things. For tropical juniper stands, there is evidence supporting that their growth is limited primarily by maximum temperatures [89,90]. However, regardless of the biome, heat waves have a negative impact on tree growth at all scales, from inhibition of photosynthesis and respiration to shedding of foliage and a decrease in the rate of apical and radial growth [91]. Of course, this phenomenon often occurs simultaneously with atmospheric and/or soil drought, resulting in mutually exacerbating water and heat stress [89,91,92,93]. This relationship is confirmed by the co-occurrence of climatic deviations observed in this study (hot and dry/cool and wet), leading to extremely slow/fast growth, respectively. One of the mechanisms linking atmospheric drought to a heat wave and, consequently, stomata closure and inhibition of transpiration and photosynthesis is the exponential dependence of the vapor pressure deficit (VPD) on air temperature [94]. This means that VPD becomes extremely volatile during the hottest months and explains the disproportionally strong response of tree growth to maximum temperature in hot climates and its persistence even in areas with high access to soil moisture (sites Be and IA in this study). Nevertheless, note that during seasonal generalization of climatic series, the response to precipitation is more pronounced than on a monthly scale, due to the accumulation of a long-term unidirectional effect of precipitation, including winter precipitation as an additional source of moisture during snowmelt [95,96]. The usage of SPEI instead of precipitation tends to increase correlation values further without a shift in seasonality. Therefore, it can be concluded that despite a moderately pronounced and complex climatic response, conifer trees of the more xeric semiarid stands of Kazakhstan are suitable for the reconstruction of climate indices associated with drought, especially those depending on both temperature and precipitation (such as SPEI or Palmer drought severity index, PDSI), on a seasonal or annual scale.

The shift in the ending of the climatic response in the current and previous growing season to earlier dates, observed in the subtropical zone, is most likely associated with the suppression of growth and inhibition of photosynthesis in trees during the extremely dry season, starting from July. Deceleration of tree cambial activity and even shifts in its timing in semiarid regions due to lack of moisture were previously described in direct observations of the seasonal kinetics of xylogenesis and from other data, but mainly for the Mediterranean climate and temperate latitudes [48,97,98,99,100,101]. In contrast to the continental subtropics, in the temperate climate with a more evenly distributed moisture deficit during the warm season, active photosynthesis and accumulation of assimilates in evergreen trees can continue until September–October [102,103,104]. Thus, the shifts in the seasonality of the response along the transect are associated both with the earlier onset of vegetation in warm climates and with the limitation of the xylogenesis seasonal timeframe by a sharp drop in precipitation at the end of the growing season.

## 5. Conclusions

Filling the existing gap in dendroclimatic research in Kazakhstan, Central Asia, we found evidence supporting the hypothesis of heat stress expressed in maximal temperatures of the previous and current growth season being the most persistent factor limiting tree growth on the semiarid boundary of conifer forests in hot climates. Heat impact is less dependent on local habitat humidity, tree species, or seasonal distribution of precipitation in comparison to drought stress, but they can exacerbate each other when co-occurring. Such a climatic response makes the lower/southern forest fringes particularly vulnerable to the current rapid warming in the region. This should be taken into account when planning measures to protect and increase the forested area in the country. We suggest the following directions for future investigations within the study region: (1) increasing the scope of dendroclimatic studies to cover the full range of environmental conditions and forest ecosystems; (2) comparative analysis of tree growth in closely spaced habitats (to ensure common climatic conditions) with contrasting water availability and/or insolation; (3) analysis of the long-term trends and low/frequency variations in tree growth in response to climatic changes; (4) experiments on heat-protection measures in recently/currently reforested areas and urban tree vegetation.

## Figures and Tables

**Table 1:** Location of the sampling sites.

Sampling Site	Species ^1^	Coordinates		
		Latitude (N)	Longitude (E)	Elevation, m a.s.l.
Bayanaul (Ba)	PISY	50°49.58′	75°42.30′	555
Shalday (Sh)	PISY	51°55.33′	78°43.72′	168
Beskaragay (Be)	PISY	50°48.04′	79°40.70′	238
Zhongar-Alatau (ZA)	PCSH	45°27.04′	80°27.20′	1190
Ile-Alatau (IA)	PCSH	43°10.30′	77°00.94′	1377
Sayram-Ugam (SU)	JUSE	42°16.41′	70°38.24′	2000

^1^ PISY—Pinus sylvestris, PCSH—Picea schrenkiana, JUSE—Juniperus seravschanica.

**Table 2:** Statistics of local tree-ring width chronologies. Sampling sites: Ba—Bayanaul, Sh—Shalday, Be—Beskaragay, ZA—Zhongar-Alatau, IA—Ile-Alatau, and SU—Sairam-Ugam.

Statistics	Sampling Sites					
	Ba	Sh	Be	ZA	IA	SU
**Sample**						
no. of trees/cores	20/20	23/23	23/23	25/25	29/29	12/12
cover period, years	1852–2017	1916–2017	1827–2017	1938–2019	1896–2019	1913–2019
total length, years	166	102	191	82	124	107
total no. of measured rings	1438	2136	2382	1264	1893	814
no. of missing rings	1	1	4	0	0	0
mean TRW, mm	1.255	2.481	1.247	2.547	2.178	1.846
**Standard chronologies**						
*SD*	0.464	0.339	0.401	0.175	0.194	0.175
*sens*	0.288	0.240	0.219	0.146	0.181	0.176
*r-bar* *	0.525	0.561	0.537	0.508	0.497	0.367
*ar*_−1_	0.742	0.624	0.766	0.518	0.369	0.263
*EPS* > 0.85, years	1946–2017	1941–2017	1913–2017	1963–2019	1930–2019	1961–2019

*SD*, standard deviation; *sens*, mean sensitivity coefficient; *r-bar*, inter-series correlation coefficient; *ar*_−1_, first-order autocorrelation; *EPS*, expressed population signal. * Statistics *r-bar* and *EPS* were calculated for moving windows of the 40-year width (limited as half of the shortest chronology) and the 1-year step; then, *r-bar* was averaged.

**Table 3:** Correlation coefficients between local standard tree-ring width chronologies, calculated over respective overlap periods after cut-off by criterion *EPS* > 0.85 (Ba—Bayanaul, Sh—Shalday. Be—Beskaragay, ZA—Zhongar-Alatau, IA—Ile-Alatau, and SU—Sairam-Ugam).

Sampling Sites	Ba	Sh	Be	ZA	IA
**Sh**	**0.42**				
**Be**	0.19	**0.50**			
**ZA**	0.11	0.13	0.00		
**IA**	0.07	0.20	0.16	**0.55**	
**SU**	0.02	−0.12	−0.03	0.22	0.05

Marked (bold) correlations are significant at *p* < 0.05.

**Table 4:** Seasonal climatic variables and their deviations for pointer years (years with the highest/lowest tree-ring width indices) at each sampling site (Ba—Bayanaul, Sh—Shalday, Be—Beskaragay, ZA—Zhongar-Alatau, IA—Ile-Alatau, and SU—Sairam-Ugam). Climatic variables are average maximal temperature for previous (prev_Tmax) and current growing season (curr_Tmax), sum of precipitation for previous (prev_P) and current growing season (curr_P), annual sum of precipitation (year_P), and average SPEI (year_SPEI).

Variable	Characteristics	Sampling Sites					
		Ba	Sh	Be	ZA	IA	SU
**prev_Tmax**	months	Jul *–Sep *	Jul *–Sep *	Aug *–Oct *	Jun *–Aug *	Jun *–Aug *	Jul *–Aug *
	*r*	**−0.500**	**−0.456**	**−0.389**	**−0.459**	**−0.400**	**−0.371**
**curr_Tmax**	months	May–Aug	May–Aug	May–Aug	May–Aug	Jun–Jul	Jun–Jul
	*r*	**−0.376**	**−0.387**	−0.165	**−0.440**	−0.180	**−0.326**
**prev_P**	months	Jul *–Sep *	Jul *–Sep *	Jul *–Oct *	Jun *–Sep *	Jun *–Oct *	Jul *–Aug *
	*r*	**0.466**	**0.413**	0.169	**0.461**	**0.493**	0.210
**curr_P**	months	May–Jul	May–Jul	May–Jul	Apr–Aug	Apr–Jul	May–Jul
	*r*	**0.400**	**0.315**	0.142	**0.385**	0.194	**0.255**
**year_P**	months	Aug *–Jul	Jul *–Jun	Jul *–Jun	Jun *–May	Jun *–May	Jul *–Jun
	*r*	**0.465**	**0.367**	0.101	**0.477**	**0.406**	**0.287**
**year_PDSI**	months	Aug *–Jul	Jul *–Jun	Jul *–Jun	Jun *–May	Jun *–May	Jul *–Jun
	*r*	**0.402**	**0.364**	0.154	**0.490**	**0.471**	**0.255**

*r*, correlation coefficient between the climatic variable and TRW chronology. Marked (bold) correlations are significant at *p* < 0.05. Asterisks (*) mark months of the previous year.

**Table 5:** Pointer years (years with the highest/lowest tree-ring width indices within cover period of climatic series) at each sampling site (Ba—Bayanaul, Sh—Shalday, Be—Beskaragay, ZA—Zhongar-Alatau, IA—Ile-Alatau, and SU—Sairam-Ugam).

Pointer Years	Sampling Sites					
	Ba	Sh	Be	ZA	IA	SU
**Positive (max TRW indices)**	**1947**, 1948, 1961, **1993**, 1995	1979, **1993**, 1994, 1995, *1996*	1913, **1970**, 1971, **1972**, 1995	**1970**, **1993**, *2003*, 2004, **2005**	1933, 1934, 1935, **1947**, 1988	**1970**, **1972**, 2002, **2005**, 2006
**Negative (min TRW indices)**	1955, 1956, 1967, 1999, 2012	1952, 1953, 1955, *2003*, 2012	1927, 1955, 1956, **1957**, 1958	1977, *1996*, 1998, 2008, 2015	1940, 1944, 1945, **1957**, 2008	1963, 1980, 1984, 2014, 2019

Pointer years common for two or more sites are underlined; pointer years common between species are bold; pointer years with varying signs are in italics.

**Figure A2:**
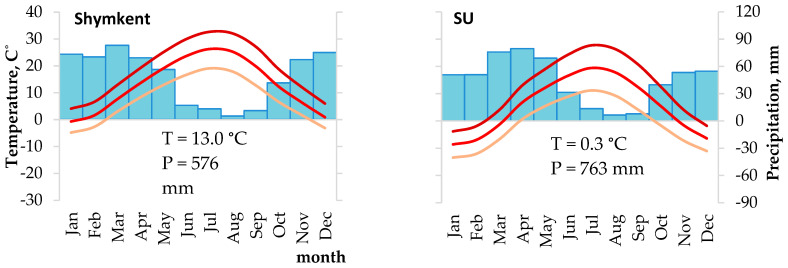
Average monthly climatic characteristics for geographic grid cell where the sampling site Sairam-Ugam (SU) is located (CRU TS 1901–2020), and at the closest meteostation in Shymkent City (42°19′ N 69°37′ E, 402 m a.s.l.; 1981–2010; http://www.pogodaiklimat.ru/climate/38328.htm; accessed on 10 April 2023) on plains to the west of that area. Lines represent maximum (dark), mean (medium), and minimum (light) temperatures; bars represent precipitation. Note that despite severe differences in the mean values of both temperatures and precipitation, their seasonal curves are remarkably similar between plains and mountains. Since the sampling site is located in the foothills on the western side of the grid cell, we should expect the mean values of temperature and precipitation to be in between the two presented diagrams.

## Data Availability

The data presented in this study are available on reasonable request from the corresponding authors.
